# Impact of experiencing multiple vulnerabilities on fetal growth and complications in women with hyperglycemia in pregnancy

**DOI:** 10.1186/s12884-023-06048-9

**Published:** 2023-10-18

**Authors:** Helene Bihan, Charlotte Nachtargeale, Eric Vicaud, Meriem Sal, Narimane Berkane, Sara Pinto, Sopio Tatulashvili, Marion Fermaut, Lionel Carbillon, Emmanuel Cosson

**Affiliations:** 1 Department of Endocrinology-Diabetology-Nutrition, AP-HP, Avicenne Hospital, Paris 13 University, CRNH-IdF, CINFO, Université Sorbonne Paris Nord Bobigny, France; 2 Laboratoire Educations Et Promotion de La Santé, LEPS, Université Sorbonne Paris Nord Bobigny, UR3412 Villetaneuse, France; 3 AP-HP, Unité de Recherche Clinique St-Louis-Lariboisière, Université Denis Diderot, 75009 Paris, France; 4 Unit of Endocrinology Diabetology Nutrition, AP-HP, Jean Verdier Hospital, CINFO, CRNH-IdF, Paris 13 University, Sorbonne Paris Cité, Bondy, France; 5 Department of Obstetrics and Gynecology, AP-HP, Jean Verdier Hospital, Paris 13 University, 93143 Sorbonne Paris Cité, Bondy France; 6 Equipe de Recherche en Epidémiologie Nutritionnelle (EREN), Inserm (U1153), Université Paris 13, COMUE Sorbonne-Paris-Cité, Inra (U1125), Centre d’Epidémiologie Et Statistiques Paris Cité, 93017 CnamBobigny, France

**Keywords:** Hyperglycemia in pregnancy, Small-for-gestational-age infant, Large-for-gestational-age infant, Psychosocial deprivation, Food insecurity, Language proficiency

## Abstract

**Background:**

In women with hyperglycemia in pregnancy living in France, psychosocial deprivation is associated with both earlier and greater exposure to the condition, as well as poorer maternofetal prognosis. We explored the impact of this and two other socioeconomic vulnerability indicators—food insecurity and poor language proficiency—on adherence to prenatal care and maternal and fetal outcomes.

**Methods:**

In a socially deprived suburb of Paris, we selected women who delivered between 01/01/2012 and 31/12/2018 and received care (nurse, dietician, diabetologist evaluation, advice, regular follow-up to adjust insulin doses if requested) for hyperglycemia in pregnancy. We analyzed the associations between individual psychosocial deprivation, food insecurity, French language proficiency (variables assessed by individual questionnaires) and fetal growth (main outcome), as well as other core maternal and fetal outcomes.

**Results:**

Among the 1,168 women included (multiethnic cohort, 19.3% of whom were Europeans), 56%, 17.9%, and 27.5% had psychosocial deprivation, food insecurity, and poor French language proficiency, respectively. Forty-three percent were prescribed insulin therapy. Women with more than one vulnerability had more consultations for diabetes. The rates for small (SGA), appropriate (AGA), and large-for-gestational-age (LGA) infant were 11.4%, 76.5% and 12.2%, respectively. These rates were similar in women with and without psychosocial deprivation, and in those with and without food insecurity. Interestingly, women with poor French language proficiency had a higher odds ratio of delivering a small- or large-for-gestational age infant than those with good proficiency.

**Conclusion:**

We found similar pregnancy outcomes for women with hyperglycemia in pregnancy living in France, irrespective of whether or not they had psychosocial deprivation or food insecurity. Optimized single-center care with specialized follow-up could contribute to reduce inequalities in maternal and fetal outcomes in women with hyperglycemia in pregnancy.

**Supplementary Information:**

The online version contains supplementary material available at 10.1186/s12884-023-06048-9.

## Background

The pooled global standardized prevalence of gestational diabetes mellitus (GDM) was estimated at 14.0% by Wang et al. using data from studies conducted between 1990–2020 [1]. In terms of regional values, standardized prevalence in Europe was estimated at 7.8% [1]. In metropolitan France, the prevalence of hyperglycemia in pregnancy (HIP) increased from 6.7% to 13.6% between 2010 and 2019 [2].

Low socioeconomic status is associated with a higher prevalence of hyperglycemia in pregnancy (HIP) [3, 4]. Furthermore, previous studies have shown an association between women with low socioeconomic and/or educational status and adverse pregnancy outcomes, specifically a higher risk of postpartum maternal and neonatal hospitalization, premature delivery, small-for-gestational-age (SGA) infant, large-for-gestational-age (LGA) infant, stillbirth, and neonatal death [5, 6].

However, few studies to date have focused on the relationship between low socioeconomic status and maternal and pregnancy outcomes in women with HIP (including early-diagnosed gestational diabetes mellitus (eGDM), gestational diabetes mellitus (GDM), and diabetes in pregnancy (DIP)). In one socially disadvantaged suburb in Paris, France, women who were most psychosocially deprived had a higher odds ratio of insulin therapy, LGA infant, and dystocia than those who were least psychosocially deprived [7]. In California, having a low education level was independently associated with a higher odds ratio of macrosomia [8]. In a small Dutch cohort, while no association was found between living in a low socioeconomic status area and adverse pregnancy outcomes, the burden of diabetes was associated with a poorer pregnancy prognosis [9].

Other vulnerabilities, such as food insecurity and poor language proficiency, may negatively influence adherence to prenatal care and pregnancy outcomes. Household food insecurity is defined as the financial inability to obtain enough food for a healthy diet. Prevalence in the general populations of the USA and France is estimated at between 11 and 16% [10, 11]. Food insecurity is associated with a higher prevalence of HIP, higher gestational weight gain, and more pregnancy-related complications [12, 13]. It may also be a risk factor for SGA infant, through lower fruit and vegetable consumption [14, 15]. Poor language proficiency, an issue faced by many migrant persons, hampers effective chronic disease management in several domains including healthcare use, patient-provider communication, and healthcare processes [16]. Although some studies in the US evaluated the impact of language proficiency on both maternal and neonatal outcomes [17, 18], to date, no study has explored this issue in women with HIP.

In this context, we aimed to evaluate the association between socioeconomic vulnerability and various HIP-related pregnancy outcomes in women from various ethnicities living in a socially deprived area in France. Three vulnerability indicators were used as follows: psychosocial deprivation (using the French-based EPICES psychosocial score), food insecurity, and French language proficiency. We hypothesized that these three socioeconomic factors were associated with poor pregnancy outcomes, especially LGA infant, through difficulties in adhering to dietary advice, to follow-up, and to insulin treatment during pregnancy.

## Methods

**Fig. 1:**
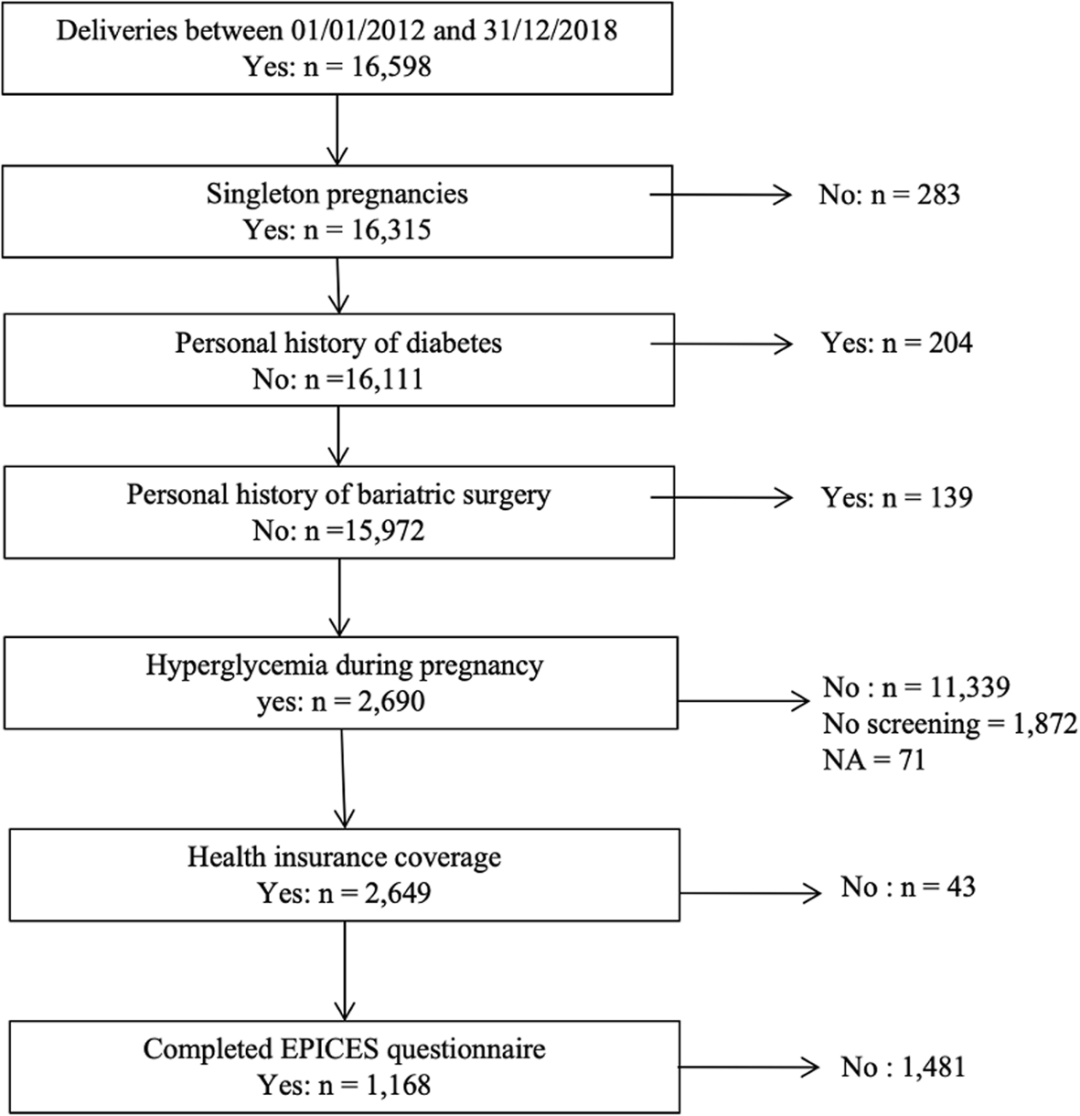
Flow chart

The data used came from Jean Verdier hospital (located in the Paris area), where the authors are based. The hospital’s Obstetrics and Gynecology department has a neonatology unit and an inpatient unit for high-risk pregnancies. Between 01/01/2012 and 31/12/2018, there were a total of 16,598 deliveries in Jean Verdier (Fig. 1: Flow chart). For the present analysis, only women with a singleton pregnancy and HIP were included. Twins and triplets were excluded to avoid possible biases induced by lower weight gain and a higher risk of complications [19]. In Jean Verdier, the midwife present at the delivery routinely and prospectively records all data for the woman giving birth.

Screening for HIP is performed in the Obstetrics and Gynecology department in accordance with the International Association of Diabetes Pregnancy Study Group (IADPSG) / World Health organization (WHO) criteria for HIP diagnosis [20, 21]. In general, diagnosis happens in three steps. First, Fasting Plasma Gluocse (FPG) is measured before 22 weeks of gestation (WG). Women with an FPG level between 5.1 and 6.9 mmol/L are diagnosed with early gestational diabetes mellitus (eGDM), while those with an FPG ≥ 7.0 mmol/L are diagnosed with diabetes in pregnancy (DIP). Second, if the FPG is normal, a 75 g oral glucose tolerance test (OGTT) is performed at 22 WG. Women with an FPG 5.1–6.9 mmol/L and/or one hour post-glucose (1 h-PG) ≥ 10.0 mmol/L and/or two hours post-glucose (2 h-PG) 8.5–11.0 mmol/L are diagnosed with GDM, whereas those with an FPG ≥ 7.0 and/or 2-h PG ≥ 11.1 mmol/L are diagnosed with DIP. Third, another screening is recommended for women not diagnosed with HIP at 22 WG, if hydramnios or macrosomia are present during an ultrasound scan [22]. Accordingly, we use three glycemic status categories after 22 weeks: early GDM (eGDM), GDM, and DIP.

The monitoring and management procedures for women with HIP in Jean Verdier hospital are described in detail elsewhere [21]. Obstetrical care at the hospital follows French recommendations [22]. Specifically, all women diagnosed with HIP who have healthcare cover are promptly referred to our multidisciplinary team (comprising a diabetologist, an obstetrician, a midwife, a dietician, and a nurse educator) for outpatient care. This team provides women with tailored dietary advice, including physical activity support, and instructions on how to perform self-monitoring of blood glucose levels six times a day. Thereafter, patients are seen by the diabetologist every 2–4 weeks. They receive insulin therapy when their fasting and 2-h postprandial glucose levels are above 5.3 and 6.8 mmol/L, respectively, as per French guidelines [22]. Antenatal visits are scheduled every 2–4 weeks until 34 weeks and weekly thereafter with cardiotocogram and amniotic fluid volume assessment. The multidisciplinary team has also set up a tailored education program for HIP care (pictorial tools, culturally appropriate actions, dedicated nurses) and provides very close monitoring. Translators are available if needed.

For the present study, the exclusion criteria were as follows: delivering twins or triplets, a personal history of diabetes, a history of bariatric surgery, no HIP screening, no healthcare cover, and missing EPICES questionnaire.

### Data collected and assessment of vulnerabilities

Baseline characteristics included age, body mass index (BMI), smoking status before pregnancy, parity, history of familial diabetes, hypertension, previous HIP, macrosomia or fetal death, and sociodemographic factors. The latter included occupation, self-reported region of origin, healthcare cover, and our three socioeconomic vulnerability indicators (i.e., psychosocial deprivation, food insecurity, and French language proficiency). Participants selected their region of origin from a list of world regions provided by the study investigator. The decision to collect data on region of origin was based on observed ethnic disparities in maternal and fetal health outcomes [23].

In France, healthcare is covered by i) social security (standard health insurance, complemented by private health cover for most of the population), ii) universal health protection for low-income persons, iii) complementary universal health protection for the poorest persons (instead of private health cover), and iv) state medical aid for those who are living in France illegally. Some people have no healthcare cover, either because they do not ask for it or because they have just immigrated.

The three socioeconomic vulnerability indicators were evaluated during the first day of diabetes care. A translator was present to help women with poor proficiency in French.

First, psychosocial deprivation was measured using the 11-item French-based EPICES questionnaire. In English, EPICES stands for Psychosocial Deprivation and Health Inequalities in Health Centers. The questionnaire items focus on various aspects of socioeconomic conditions and family environment (see Additional file) [24]. For the present study, we used an EPICES score > 30.17 to define psychosocial deprivation [25].

Second, food insecurity was assessed using a single question with four possible response options as follows: “Which of these statements best describes the food eaten in your household? i) Enough of the kinds of food you want to eat; ii) Enough but not always the kinds of food you want to eat; iii) Sometimes not enough to eat; iv) Often not enough to eat”. This question is used in the U.S. Household Food Security Survey Module by the USA Department of Agriculture to measure food insecurity [10]. Participants were categorized as having food insecurity if they answered options iii) or iv).

Third, participants were asked how well they spoke French. There were three propositions: poor, moderate, or good proficiency.

### Study outcomes

The primary outcome was neonatal weight at birth according to gestational age, classified into three categories: SGA, appropriate-for-gestational-age (AGA), and LGA infant. LGA and SGA infant were defined as a birth weight greater than the 90^th^ percentile and lower than the 10^th^ percentile for the standard French population, respectively [26].

The secondary outcomes were HIP-related events, categorized into maternal and neonatal complications [27]. For maternal outcomes, we considered hypertensive disorders of pregnancy, pre-eclampsia and cesarean sections. Hypertensive disorders include hypertension before pregnancy, gestational hypertension and pre-eclampsia. Gestational hypertension was defined by the onset of hypertension (blood pressure ≥ 140 mmHg systolic or ≥ 90 mmHg diastolic) at or after 20 WG, in the absence of proteinuria and without biochemical or hematological abnormalities [28]. When earlier blood pressure values were unknown, we considered that hypertensive disorder was not present. Pre-eclampsia was defined as having a blood pressure ≥ 140/90 mmHg for two measurements four hours apart, and proteinuria of at least 300 mg/24 h or a 3 + level with dipstick testing in a random urine sample, and/or evidence of maternal acute kidney injury, liver dysfunction, neurological features, hemolysis or thrombocytopenia, and fetal growth restriction [28]. Caesarean sections were defined as selective and emergency caesarean sections before or during delivery.

For neonatal outcomes, we considered shoulder dystocia, prematurity, neonatal hypoglycemia, neonatal death and stillbirth [29]. Shoulder dystocia was defined as the use of obstetrics maneuvers. Prematurity was defined as occurring before 37 WG, and neonatal hypoglycemia as at least one blood glucose measurement under 2 mmol/L during the first two days of life. Finally, we also considered stillbirth and neonatal death (in the first 24 h of life).

### Explanatory variables: Care for HIP during pregnancy

For the present study, we collected data on two habits (smoking status and fruit and vegetable consumption), and on healthcare during pregnancy. During the dietary survey at HIP care initiation, all women answered the following question “Do you eat fruit and/or vegetables every day?”. Data collected on healthcare included gestational weight gain (GWG), insulin therapy (yes or no), the time between their most recent HIP outpatient visit and their first related prescription of insulin (days), the number of consultations with a diabetologist between HIP diagnosis and delivery (number), whether hospitalization was required for uncontrolled diabetes or for pregnancy complications (percentage of women requiring hospitalization, number of hospitalizations for each woman), and hospitalization duration (days). GWG was calculated as the weight just before delivery minus the self-reported pre-pregnancy weight. At Jean Verdier hospital, women receive insulin therapy (no other treatment is used in France for HIP) when pre-prandial and/or 2-h post-prandial glucose levels are greater than 5.0 and/or 6.7 mmol/L, respectively [22].

### Statistical analyses

We collected all the study data with the help of other physicians, and a research study technician created the database. EC and EV ensured the accuracy of the database contents. Descriptive statistics were used to describe the entire population in terms of sociodemographic parameters, medical history, and glycemic figures. Baseline continuous variables were expressed as mean ± standard deviation. Categorical variables were expressed as frequencies (percentages).

Missing data were not replaced. With regard to French language proficiency level, we created a binary variable: poor combined ‘poor’ and ‘moderate’ proficiency, and good reflected ‘good’ proficiency.

Student’s t-test was used to compare continuous variables. The Chi-squared or Fisher’s-exact test was used for categorical variables, as appropriate. To explore the possible presence of selection bias, the baseline characteristics of the women included were compared with those not included.

Patient characteristics were compared for the three vulnerability indicators (i.e., psychosocial deprivation, food insecurity, and French language proficiency).

The three categories of neonatal weight at birth according to gestational age (i.e., primary outcome) were compared between the three secondary outcomes), as well as vulnerability indicators. We then analyzed maternal and neonatal outcomes (i.e., habits (i.e., smoking status and fruit and vegetable consumption) and care during pregnancy (i.e., explanatory variables) according to the same three indicators. To evaluate HIP-related events, we performed an exploratory analysis where we created a composite criterion that included pre-eclampsia, LGA infant, and shoulder dystocia. However, as pre-eclampsia and shoulder dystocia were rare, this analysis was not retained as it did not add any new information.

We analyzed patient characteristics associated with the three categories of neonatal weight at birth and performed a multinomial logistic regression with AGA as the reference category. Variables associated with the outcomes SGA or LGA infant (i.e., as AGA was the reference) with a *p*-value < 0.10 in the univariate analysis were included in the multinomial logistic regression. We did not include tobacco as the prevalence of smoking was very low or inexistent.

A *p*-value < 0.05 was considered statistically significant. All data analyses were performed using R software, version 3.6.3.

## Results

### Population characteristics

Of the potential 2,649 participants, 1,168 completed the EPICES questionnaire and answered the two questions on food insecurity and French language proficiency. These women were included in the present analysis. They were similar to non-included women except that a lower percentage were unemployed (63.4 vs. 68.4%, respectively; *p* = 0.01) (Additional Table).

Of those included, 31.2% had eGDM, 62.3% GDM and 6.5% DIP. Mean age was 33 years, 27% had obesity and 34.2% reported a family history of diabetes (Table 1). With regard to sociodemographic characteristics, 36.6% were employed. Almost one-fifth (19.3%) were born in Europe, 36% in North Africa, 15% Sub-Saharan Africa, 17.3% in South Asia, and 7.5% elsewhere. Just over half (55.6%) had standard health insurance. In terms of the three socioeconomic vulnerability indicators studied, 56% suffered from psychosocial deprivation, 17.9% reported food insecurity, and 27.5% reported poor French language proficiency.

**Table 1:** Characteristics of the cohort, medical history and glycemic figures, and sociodemographic parameters (1,168 women delivered between 01/01/2012 and 31/12/2018 in Jean Verdier University hospital, France)

Parameters	N	Total *N* = 1,168	No psychosocial deprivation^a^ *N* = 515 (44%)	Psychosocial deprivation^a^ *N* = 653 (56%)	*P*
**Medical history and glycemic figures**					
**EPICES Score**	1,168	36.1 ± 21.2	16.7 ± 8.2	51.3 ± 15.0	**0.00**
Age (years)	1,168	32.7 ± 5.4	32.67 ± 5.4	32.7 ± 5.4	1.00
Body mass index (kg/m2)	1,168	27.0 ± 5.4	27.0 ± 5.5	27.0 ± 5.3	0.93
Obesity	1,168	314 (27.0)	14.9 (28.9)	16.5 (25.5)	0.19
Hypertension before pregnancy	1,168	16 (1.4)	5 (1.0)	11 (1.7)	0.30
Family history of diabetes	1,168	400 (34.2)	197 (38.3)	203 (31.1)	**0.01**
Smoking before pregnancy	1,168	97 (8.3)	56 (10.9)	41 (6.3)	**0.00**
Parity (number of children)	1,168	2.3 ± 1.3	2.1 ± 1.1	2.5 ± 1.4	**0.00**
**History of HIP**	1,168				0.60
1^st^ child		368 (31.5)	189 (36.7)	179 (27.4)	
no		601 (51.5)	235 (45.6)	366 (56.0)	
yes		199 (17.0)	91 (17.7)	108 (16.5)	
**History of macrosomia**	1,168				0.64
1^st^ child		368 (31.5)	189 (36.7)	179 (27.4)	
no		731 (62.6)	298 (57.9)	366 (56.0)	
yes		69 (5.9)	28 (5.4)	108 (16.5)	
**History of fetal death**	1,168				0.84
1^st^ pregnancy		218 (18.7)	105 (20.4)	113 (17.3)	
no		911 (78.0)	393 (76.3)	518 (79.3)	
yes		39 (3.3)	17 (3.3)	22 (3.4)	
**Screening for HIP before 22 WG**					0.31
Fasting plasma glucose (mmol/L)	776	5.2 ± 0.8	5.2 ± 0.8	5.2 ± 0.8	
**OGTT at 22 WG and after**					
Fasting plasma glucose (mmol/L)	811	5.1 ± 0.7	5.0 ± 0.6	5.2 ± 0.8	**0.01**
1H-post OGTT glucose (mmol/L)	752	9.5 ± 2.0	9.5 ± 1.8	9.5 ± 2.1	0.83
2H-post OGTT glucose (mmol/L)	756	8.2 ± 1.9	8.2 ± 1.8	8.1 ± 2.0	0.54
**HIP status**	1,168				0.67
eGDM		364 (31.2)	164 (31.8)	200 (30.6)	
GDM		728 (62.3)	321(62.3)	407 (62.3)	
DIP		76 (6.5)	30 (5.8)	46 (7.0)	
**Sociodemographic parameters**					
**Employment**					**0.00**
Working	1,166	427 (36.6)	274 (53.3)	153 (23.5)	
**Self-reported region of origin**	1,166				**0.00**
Sub-Saharan Africa		179 (15.4)	40 (7.8)	139 (21.3)	
North Africa		420 (36.0)	212 (41.2)	208 (31.9)	
Other		88 (7.5)	41 (8.0)	47 (7.2)	
Europe		225 (19.3)	155 (30.2)	70 (10.7)	
Haiti, French overseas territories		52 (4.5)	20 (3.9)	32 (4.9)	
South Asia		202 (17.3)	46 (8.9)	156 (23.9)	
**Health insurance coverage**	799				**0.00**
Social Security		444 (55.6)	261 (71.7)	183 (42.1)	
Universal health protection		190 (23.8)	44 (12.1)	146 (33.6)	
Complementary universal health protection		93 (11.6)	54 (14.8)	39 (9.0)	
State medical aid		72 (9.0)	5 (1.4)	67(15.4)	
**Poor French language proficiency**	1,013	279 (27.5)	60 (13.1)	219 (39.5)	**0.00**
**Food insecurity**	887	159 (17.9)	24 (5.9)	135 (28.2)	**0.00**

*HIP* hyperglycemia in pregnancy, *OGTT 75* g oral glucose tolerance test, *WG* Gestational weeks, *eGDM* early-diagnosed gestational diabetes mellitus, *GDM* Gestational diabetes mellitus, *DIP* Diabetes in pregnancy

^a^Psychosocial deprivation was defined as an EPICES score > 30.17

Compared with women who did not suffer from psychosocial deprivation, those who did were less likely to have a family history of diabetes, to smoke, to work, and to have universal health protection and state medical aid (see Table 1). In contrast, they were more likely to be immigrants from Sub-Saharan Africa and South Asia, and more likely to have higher parity and higher FPG values from the OGTT test. Finally, women with psychosocial deprivation were more likely to report food insecurity and poor French language proficiency.

## Outcomes according to psychosocial deprivation status

### Primary outcome

The rates of SGA, AGA and LGA infant were 11.4%, 76.5% and 12.2%, respectively (Table 2), with a similar distribution in women with and without psychosocial deprivation, and in those with and without food insecurity. Neonatal weight at birth according to gestational age was associated with French language proficiency level (Table 3). Women reporting poor proficiency were more likely to experience abnormal fetal growth (25.3% of infants overall, specifically 11.4% SGA, and 13.9% LGA) than women reporting good proficiency (18.3% overall, specifically 10.8% of SGA, and 7.5% LGA), with *p* = 0.02 (Table 3).

**Table 2:** Determinants of fetal growth (1,168 women delivered between 01/01/2012 and 31/12/2018 in Jean Verdier University hospital, France)

**Parameters**	**Small-for-gestational-age**^**a**^ *N* = 133 (11.4%)	**Appropriate-for-gestational-age** *N* = 893 (76.5%)	**Large-for-gestational-age**^**a**^ *N* = 142 (12.2%)	**P**
**Indicators of socioeconomic vulnerability**				
**Psychosocial deprivation**	36.4 ± 21.1	36.1 ± 21.3	35.9 ± 20.7	
**French language proficiency**	30 (26.3)	228 (29.4)	21 (17.1)	**0.00**
**Food insecurity**	21 (20.8)	120 (17.8)	18 (16.2)	0.67
**Medical history and glycemic figures**				
Age (years)	32.8 ± 5.2	32.7 ± 5.3	32.5 ± 5.5	0.89
Body mass index (kg/m2)	26.1 ± 5.6	26.7 ± 5.2	29.3 ± 5.8	**0.00**
Obesity	23 (17.6)	231 (26.0)	60 (42.3)	**0.00**
Hypertension before pregnancy	2 (1.5)	12 (1.3)	2 (1.4)	0.30
Family history of diabetes	43 (32.3)	296 (33.1)	61 (43.0)	0.06
**Smoking** before pregnancy	13 (9.8)	75 (8.4)	9 (6.3)	0.09
Parity, number of children	1.9 ± 1.2	2.3 ± 1.3	2.6 ± 1.1	**0.00**
**History of HIP**				**0.00**
1^st^ child	70 (52.6)	274 (30.7)	24 (16.9)	
no	42 (31.6)	488 (54.6)	71 (50.0)	
yes	21 (15.8)	131 ((14.7)	47 (33.1)	
**History of macrosomia**				**0.00**
1^st^ child	70 (52.6)	274 (30.7)	24 (16.9)	
no	62 (46.6)	579 (64.8)	90 (63.4)	
yes	1 (0.8)	40 (4.5)	28 (19.7)	
**History of fetal death**				0.76
1^st^ pregnancy	37 (27.8)	165 (18.5)	16 (11.3)	
no	91 (68.4)	700 (78.4)	120 (84.5)	
yes	5 (3.8)	28 (3.1)	6 (4.2)	
**Screening for HIP before 22 WG**				**0.04**
Fasting plasma glucose (mmol/L)	5.0 ± 0.5	5.2 ± 0.9	5.1 ± 0.5	
**OGTT at 22 WG and after**				
Fasting plasma glucose (mmol/L)	5.0 ± 0.7	5.1 ± 0.7	5.1 ± 0.7	0.14
1H-post OGTT glucose (mmol/L)	9.8 ± 2.1	9.4 ± 2.0	9.5 ± 2.0	0.28
2H-post OGTT glucose (mmol/L)	8.5 ± 2.0	8.1 ± 1.9	8.3 ± 2.2	0.16
** HIP status**	43 (32.4)	275 (30.8)	46 (32.4)	0.93
eGDM	81 (60.9)	562 (62.9)	85 (59.9)	
GDM	9 (6.8)	56 (6.3)	11 (7.7)	
DIP				
**Gestational weight gain**	8.4 ± 4.8	9.3 ± 5.5	10.3 ± 6.2	**0.03**
**Sociodemographic parameters**				
**Employment**				
Working	50 (37.9)	327 (36.7)	50 (35.2)	0.90
**Self-reported region of origin**				**0.02**
Sub-Saharan Africa	20 (15.2)	141 (15.8)	18 (12.7)	
North Africa	35 (26.5)	310 (34.8)	75 (52.8)	
Other	10 (7.6)	72 (8.1)	6 (4.2)	
Europe	28 (21.2)	173 (19.4)	24 (16.9)	
Haiti, French overseas territories	6 (4.5)	43 (4.8)	3 (2.1)	
South Asia	33 (25.0)	153 (17.2)	16 (11.3)	
**Health insurance coverage**				**0.049**
Social Security	45 (52.3)	349 (56.7)	50 (51.0)	
Universal health protection	20 (23.3)	149 (24.2)	21 (21.4)	
Complementary universal health protection	10 (11.6)	62 (10.1)	21 (21.4)	
State medical aid	11 (12.8)	55 (8.9)	6 (6.1)	
**Indicators of socioeconomic vulnerability**				
Psychosocial deprivation	36.36 ± 21.08	36.09 ± 21.31	35.91 ± 20.76	
French language proficiency	30 (26.3)	228 (29.4)	21 (17.1)	**0.003**
Food insecurity	21 (20.8)	120 (17.8)	18 (16.2)	0.67

*HIP* Hyperglycemia in pregnancy, *OGTT 75* g oral glucose tolerance test, *WG* Gestational weeks, *eGDM* early-diagnosed gestational diabetes mellitus, *GDM* Gestational diabetes mellitus, *DIP* Diabetes in pregnancy

^a^SGA and LGA infant were defined as a birth weight lower than the 10^th^ percentile and greater than the 90^th^ percentile for the standard French population, respectively

**Table 3:** Maternal and neonatal outcomes according to vulnerability indicators (1,168 women delivered between 01/01/2012 and 31/12/2018 in Jean Verdier University hospital, France)

		**Psychosocial deprivation**^**a**^		**Food insecurity**^**b**^		**French language proficiency**^**c**^	
	Total (*n* = 1,168)	No (*n* = 515)	Yes (*n* = 653)	No (*n* = 728)	Yes (*n* = 159)	Good (*n* = 734)	Poor (*n* = 179)
**Primary outcome**							
**Fetal growth**			*(p* = *0.3)*		*(p* = *0.7)*		*(p* = ***0.02****)*
**SGA**	133 (11.4)	51(9.9)	82 (12.6)	80 (11.0)	21 (13.2)	30 (10.8)	84 (11.4)
**AGA**	893 (76.5)	397 (77.1)	496 (76.0)	555 (76.2)	120 (75.5)	228 (81.7)	548 (74.7)
**LGA**	142 (12.2)	67 [13]	75 (11.5)	93 (12.8)	18 (11.3)	21 (7.5)	102 (13.9)
**Secondary maternal outcomes**							
**Hypertensive disorders**	79 (6.8)	35 (6.8)	44 (6.7)	46 (6.3)	16 (10.1)	57 (7.8)	11 (3.9)
**Pre-eclampsia**	38 (3.3)	17 (3.3)	21 (3.2)	21 (2.9)	8 (5.0)	28 (3.8)	4 (1.4)
**Caesarian section**	304 [26]	132 (25.6)	172 (26.3)	201 (27.6)	42 (26.4)	200 (27.2)	66(23.7)
**Secondary neonatal outcomes**							
**Shoulder dystocia**	1(0.1)	1 (0.2)	0 (0)	1 (0.1)	0 (0)	1 (0.1)	0 (0.0)
**Preterm birth**	75 (6.4)	32 (6.2)	43 (6.6)	51 (7.0)	9 (5.7)	54 (7.4)	13 (4.7)
**Hypoglycemia**	23 (2.0)	10 (1.9)	13 (2.0)	16 (2.2)	3 (1.9)	12 (1.6)	7 (2.5)
**Neonatal death and stillbirth**	2 (0.2)	1 (0.2)	1 (0.2)	2 (0.3)	0 (0.0)	2 (0.3)	0 (0.0)

Comparisons between groups (with or without vulnerability) were performed with Pearson’s Chi-squared test, none was significant. *SGA *small-for-gestational-age, *AGA * Appropriate-for-gestational-age, *LGA *Large-for-gestational-age

^a^Psychosocial insecurity was defined as an EPICES score > 30.17

^b^Food insecurity was defined when participants answered: ‘sometimes not enough to eat’ (option 3) or ‘often not enough to eat’ (option 4) to the question “Which of these statements best describes the food eaten in your household? “

^c^French language proficiency was self-reported as poor, moderate, or good. We subsequently created a binary variable as follows: poor which combined ‘poor’ and ‘moderate’ proficiency, and good for ‘good’ proficiency

**Fig. 2:**
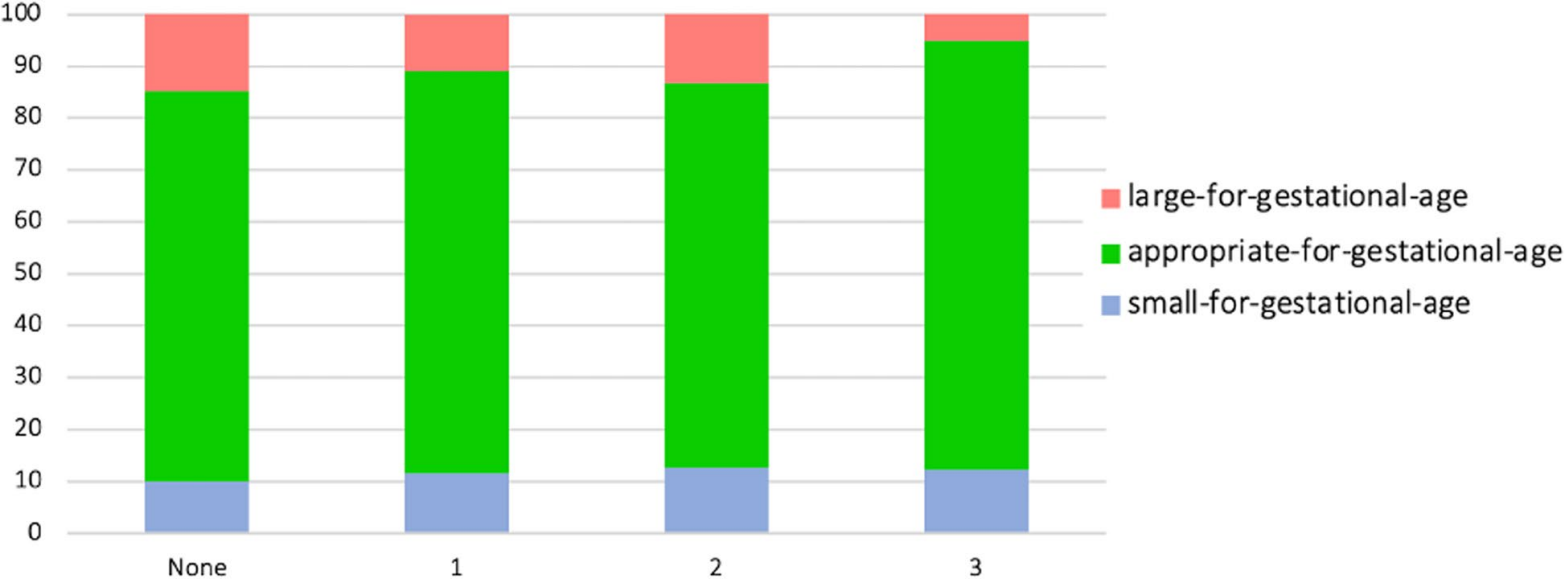
Fetal growth according to the number of socioeconomic vulnerabilities Legend: Vulnerabilities were psychosocial deprivation, food insecurity, and poor French language proficiency

In an exploratory analysis, we explored the distribution of SGA-AGA-LGA infant according to the combination of the three indicators for socioeconomic vulnerability. A total of 880 women had available data for this analysis. Figure 2 shows that women with more than one vulnerability were more likely to have an SGA or LGA infant than those with one or no vulnerability; however, these differences were not significant.

## Secondary outcomes and explanatory variables

One in seven women had secondary outcomes: pre-eclampsia (3.3%), LGA infant (12.2%) and/or an infant with shoulder dystocia (0.1%). Irrespective of the socioeconomic vulnerability indicator chosen, the rates of HIP-related outcomes were similar between each indicator category (Table 3). Participants with psychosocial deprivation or food insecurity were more likely to have an unhealthy diet than women with neither of these two vulnerability indicators.

Women with poor French language proficiency were more likely to be non-smokers, and less likely to have an unhealthy diet than those reporting good proficiency. Weight gain was lower in women who had food insecurity than in those who did not (8.0 ± 5.3 vs. 9.4 ± 5.6 kg, respectively, *p* = 0.01).

Insulin therapy was prescribed to nearly half the participants (43.3%). This percentage was similar for persons with and without each studied vulnerability. No difference was observed in the time before initiating insulin after the first HIP outpatient visit.

Having food insecurity or poor French language proficiency was associated with a higher number of consultations (4.0 ± 2.6 in those with food insecurity vs. 3.5 ± 2.5 in those without, *p* = 0.04; 3.9 ± 2.7 in women with poor French language proficiency vs. 3.4 ± 2.4 in those with good proficiency, *p* = 0.01). The mean number of hospitalizations was lower among women who had psychosocial deprivation compared to those who did not Table 4.

**Table 4:** Habits and care for hyperglycemia in pregnancy according to each indicator of psychosocial vulnerabilities

		**Psychosocial deprivation**			**Food insecurity**			**French language proficiency**		
	Total	No	Yes	*p*	No	Yes	*p*	Good	Poor	*p*
Smoking	52 (4.5)	25 (4.9)	27 (4.1)	0.55	33 (4.5)	8 (5.0)	0.79	43 (5.9)	3 (1.1)	**0.00**
Unhealthy diet ^a^	224 (19.7)	70 (13.7)	154 (24.5)	**0.00**	130 (18.1)	44 (28.4)	**0.01**	156 (21.6)	39 (14.6)	**0.01**
Weight gain (kg)	9.3 ± 5.5	9.6 ± 5.4	9.2 ± 5.7	0.23	9.4 ± 5.6	8.0 ± 5.3	**0.01**	9.3 ± 5.6	8.7 ± 5.2	0.14
Insulin therapy	506 (43.3)	211 (41.0)	295 (45.2)	0.15	305 (41.9)	74 (46.5)	0.28	304 (41.4)	132 (47.3)	0.09
Time before insulin initiation (days)	2.5 ± 3.6	2.5 ± 3.7	2.4 ± 3.6	0.72	2.4 ± 3.4	2.8 ± 3.9	0.45	2.6 ± 3.7	2.4 ± 3.7	0.73
Number of consultations	3.5 ± 2.5	3.4 ± 2.4	3.5 ± 2.6	0.23	3.5 ± 2.5	4.0 ± 2.6	**0.04**	3.4 ± 2.5	3.9 ± 2.7	**0.01**
Hospitalization after 22 WG ^b^	346 (29.6)	169 (32.8)	177 (27.1)	**0.03**	229 (31.5)	50 (31.4)	0.99	230 (31.3)	72 (25.8)	0.09
Duration of hospitalization (days)	4.3 ± 4.5	4.0 ± 4.7	4.6 ± 4.2	0.19	4.2 ± 4.6	4.5 ± 3.9	0.71	4.5 ± 4.9	3.8 ± 3.1	0.25

Data are number (percentage) or n ± deviation standard

^a^ Unhealthy diet means “No daily consumption of fruit/vegetables/ wholegrain bread”

^b^ Hospitalizations after 22 WG included hospitalizations for diabetes and other reasons

### Other parameters associated with fetal growth

As described in Table 2, the factors associated with LGA infant were obesity, higher parity, a history of HIP, a history of macrosomia, a higher FPG level before 22 WG, and greater gestational weight gain during pregnancy.

Women of South Asian origin were more likely to have an SGA infant (25% vs. 11.3% for SGA infant).

Multinomial logistic regression variables included BMI, parity, gestational weight gain, HIP status, macrosomia history, ethnicity, health insurance cover, French language proficiency, and newborn’s sex. Of these, parity (odds ratio (OR) 0.76 (95% interval confidence 0.59–0.99), *p* = 0.04), South-Asian ethnicity (OR 2.97 (1.16–7.58), *p* = 0.02), and poor French language proficiency (OR 0.36 (0.15–0.84), *p* = 0.02) were independent predictors of SGA infant. Weight gain (OR 1.04 (1.00–1.08), *p* = 0.045), macrosomia (OR 5.33 (2.47–11.54), *p* < 0.01), and complementary universal health protection (OR 2.35 (1.21–4.55), *p* = 0.01) were independently associated with LGA infant.

## Discussion

In this monocentric cohort, neither the maternal nor neonatal prognosis of HIP was related to psychosocial deprivation or to food insecurity. The high proportion of participants receiving insulin therapy and the large number of follow-up consultations are two factors that may explain these reassuring results. However, poor French language proficiency was associated with SGA and LGA infant. The latter result suggests the importance of a good understanding of dietary messages in HIP populations. Poor nutrition could impact fetal growth and induce placental dysfunction.

### Socioeconomic inequalities and maternal outcomes

Unlike the present work, previous studies reported poorer maternal and neonatal outcomes for the most psychosocially deprived pregnant women. The inverse care law supports the notion that “the availability of good medical care tends to vary inversely with the need for it in the population served” [30]. This concept has not been studied widely in the context of pregnant women. The burden of psychosocial deprivation on pregnant women and its association with an unhealthy diet may explain the higher risk of severe pregravid obesity, higher risk of HIP and earlier diagnosis, higher gestational weight gain, lower control of glycemic figures and more frequent insulin therapy which we found in our study [4, 7, 13]. Moreover, psychosocial deprivation increases the risk of inadequate prenatal care. In one study, among 10,419 pregnancies, 23.3% of women with a low socioeconomic status either reported late prenatal care, attended fewer than 50% of planned consultations, or missed their third trimester anomaly scan [31] which screens for abnormal fetal growth [32]. Moreover, the higher the number of psychosocial vulnerabilities reported, the higher was the risk of inadequate prenatal care. Elsewhere, using a qualitative approach, Whittle et al. suggested that material need-based insecurities induce uncertainty and experiences of discrimination, leading to lower implications for health in affected women [33]. We agree with Chung’s hypothesis that a low education level may affect the pregnancy outcomes of patients with complex medical conditions [8].

### Socioeconomic inequalities and fetal growth

We found a relationship between poor French language proficiency and abnormal fetal growth. This is not surprising given that women with a low socioeconomic status and with HIP have a higher risk of LGA [8, 34] or SGA infant [35–37], or both [38].

Furthermore, in a previous study by our group, the risk of LGA infant was 50% higher among women with a low socioeconomic status in a cohort comprising 996 women (56% of whom had psychosocial deprivation) [34]. In China, Chung et al*.* demonstrated a 35% higher odds ratio of LGA infant (OR 1.35 (1.09–1.70)) in women with a middle-school level of education compared to college-educated participants [8].

In contrast, in another Chinese cohort, the most psychosocially deprived participants had a higher odds ratio of SGA infant than the least deprived (OR 1.63 (95% CI 1.29–2.08) for the 4^th^ quartile compared to the 1^st^) [39]. In the USA, Harper et al. reported that women whose gestational weight gain was lower than the recommended value were more likely to have an SGA infant than those whose gestational weight gain was higher than that recommended (9.1% vs. 7.3%, respectively) [37]. Recently, Jardine et al. estimated that 31.1% of fetal growth restriction could be attributed to socioeconomic inequality in England; this proportion decreased to 16.4% when adjusted for ethnic group, smoking and BMI. In their cohort, women from South Asia had a higher odds ratio of fetal growth restriction [35].

Elsewhere, Wentz et al. highlighted that area-based deprivation was more strongly associated with LGA infant than with SGA infant [38]. The same authors observed variations in the level of association depending on race/ethnic group. It is difficult to reconcile all these contrasting findings.

### Possible explanations for our encouraging results that neither psychosocial deprivation nor food insecurity affected maternal and neonatal outcomes

Here we discuss some hypotheses as to why we found similar maternal and neonatal outcomes, irrespective of psychosocial deprivation or food insecurity status. First, the monocentric characteristic of our study may have reduced disparities. Second, the rate of insulin therapy was higher in the present study (43.3%) than in a previous study by our team on psychosocial deprivation in women with HIP from the same Paris suburb (29.4%) [7]. It was also higher than in a Japanese cohort (8.6% of women with HIP (defined according to IADPSG/WHO criteria) where the mean BMI was 22.2 kg/m^2^ [40]). In other studies, this information was not reported [36, 37]. Third, most women in our sample had psychosocial deprivation, and healthcare providers are used to treating this population. Fourth, the mean number of pregnancy consultations was also relatively high, and higher in women with food insecurity and/or poor French language proficiency. This may have translated into better tailoring of insulin therapy to the individual’s needs, and therefore better pregnancy outcomes. Increasing inter-professional communication between the specialists in our hospital (obstetricians, dieticians and diabetologists) could lead to more frequent and effective follow-up of diabetes. Indeed, glycemic monitoring is not performed only by diabetologists; it involves the participation of various specialists.

### Possible explanation for the discouraging result that language proficiency negatively affected maternal and neonatal outcomes

We observed a higher rate of SGA infant in women with poor French language proficiency. A previous study in Canada highlighted the consequences of language barriers on pregnancy outcomes. Specifically, it found that non-English-speaking Asian women had a lower risk of LGA infant than English-speaking women [16]. We hypothesize that women with poor French language proficiency in our sample may have been more afraid of their diagnosis of HIP, as they would not have been able to understand the complex related vocabulary and arguments (controlled carbohydrate diet, fruit snacks, six daily glucose control tests, different fasting and postprandial goals, weight follow-up, insulin requirements, etc.), and that the consequence of this may have been an overly restrictive diet in order to avoid gaining weight [41]. In our study, the mean gestational weight gain was 8.2 ± 4.4 kg vs. 9.1 ± 5.6 kg, respectively, for women with good proficiency.

However, in the multivariable analysis (data not presented), the main factor associated with SGA infant was South Asian ethnicity, while poor proficiency was an independent protective factor. This finding mitigates our initial hypothesis that women restrict their consumption of certain food types. Furthermore, Venkatesh et al. published data on maternal and fetal outcomes for women with HIP in the USA over a period of six years. They reported a higher risk of SGA infant in persons of Asian/Pacific island origin than in white women (OR 1.84 (1.82–1.87)), after multiple adjustment [42].

### Placental dysfunction

There was a relatively high rate of pre-eclampsia (3.3% (range 1.3–5%)) in our study population, especially in women with food insecurity (5%). The high SGA infant rate in this group (13.2%) would suggest overrepresentation of fetal growth restriction, due to a possible combination of endogenic and exogenic oxidative stress with placental dysfunction, reflected in the higher rate of pre-eclampsia observed [43]. Women with psychosocial deprivation or food insecurity reported an unhealthy diet more often than those without these vulnerabilities. The consumption of fruit, vegetables, cereals and grains is necessary for fetal growth because of their selenium content (on average, 41% of total selenium intake); other sources of selenium are fish and seafood (29%), meat (23%), and egg and dairy products (20%) [44].

### Strengths and limitations

The strengths of our study include a large study sample with three socioeconomic vulnerability indicators to evaluate socioeconomic deprivation (French language proficiency level being investigated for the first time), a multi-ethnic cohort with adjustments being made for confounders, and similar prenatal care provided by specialists, diabetologists and obstetricians to all those included.

The study also has several limitations. First, as only women who provided complete data (i.e., fully completed the EPICES questionnaire, answered the question on food insecurity, and the question on French language proficiency) were included, they differed somewhat from those not included (Additional Table). Second, as French language proficiency was self-reported instead of using a reading/writing/listening test, social desirability bias cannot be excluded. Third, the EPICES psychosocial indicator was initially validated using only people from the French ethnic group. In our study, this group accounted for less than 20% of the study population. Finally, confounding bias may have been induced by the association between ethnicity and fetal growth. Most women with poor language proficiency were from south Asia; this ethnicity has already been associated with a higher risk of SGA infant [42].

## Conclusions

Socioeconomic vulnerabilities are established risk factors of poorer maternal and neonatal outcomes during pregnancy, and of higher rates of HIP. However, in our study of women with HIP, we found similar outcomes irrespective of psychosocial deprivation and food security status. Only poor French language proficiency was associated with a lower rate of AGA infant; the reasons for this are unclear. We believe that optimized comprehensive care in a single center could reduce health inequalities in women with HIP experiencing one or more socioeconomic vulnerabilities.

### Supplementary Information

**Additional file 1.**

**Additional file 2.**

## Data Availability

EC is responsible for this work and as such, had full access to all the data in the study. Moreover, he is responsible for the integrity of the data and the accuracy of the data analysis. The datasets generated during and/or analyzed in the current study are available from the corresponding author on reasonable request.

## Abbreviations

AGA: Appropriate-for-gestational-age
BMI: Body mass index
DIP: Diabetes in pregnancy
eGDM: Early-diagnosed gestational diabetes mellitus
EPICES: French Individual index of deprivation (*Evaluation de la Précarité et inégalités de santé dans les Centres d’Examens de Santé*)
GDM: Gestational diabetes mellitus
WG: Weeks of gestation
HIP: Hyperglycemia in pregnancy
IADPSG: International association of diabetes pregnancy study group
LGA: Large-for-gestational-age
OR: Odds Ratio
OGTT: 75-G oral glucose tolerance test
SGA: Small-for-gestational-age
WHO: World Health Organization
