# Supplementary material for: Impact of experiencing multiple vulnerabilities on fetal growth and complications in women with hyperglycemia in pregnancy
Source: BMC Pregnancy Childbirth. 2023 Oct 18;23:740. doi: 10.1186/s12884-023-06048-9 (PMC10585815; doi:10.1186/s12884-023-06048-9)
Supplement: Supplementary file 1 — Additional file 1. [file 12884_2023_6048_MOESM1_ESM.docx]

**Supplementary Table:** Characteristics of all women with HIP

| **Included** | No (N=1,481) | Yes (N=1,168) | P |
| --- | --- | --- | --- |
| Age (years) | 32.3±5.5 | 32.7±5.4 | 0.06 |
| No occupation | 1013 (68.4) | 739 (63.4) | **0.01** |
| **Self-reported region of origin** |  |  | 0.23 |
| Sub-Saharan Africa | 260 (17.6) | 179 (15.4) |  |
| North Africa | 523 (35.3) | 420 (36.0) |  |
| Other | 98 (6.6) | 88 (7.5) |  |
| Europe | 318 (21.5) | 225 (19.3) |  |
| Haiti, French overseas territories | 57 (3.9) | 52 (4.5) |  |
| South Asia | 224 (15.1) | 202 (17.3) |  |
| **Health insurance coverage** |  |  | 0.44 |
| Social Security | 5 (38.5) | 444 (55.6) |  |
| Universal health protection | 5 (38.5) | 190 (23.8) |  |
| Complementary universal health  protection | 2 (15.4) | 93 (11.6) |  |
| State medical aid | 1 (7.7) | 72 (9.0) |  |
| **Smoking** before pregnancy | 103 (7.0) | 97 (8.3) | 0.19 |
| Smoking during pregnancy | 66 (4.5) | 52 (4.5) | 0.99 |
| Body mass index (kg/m2) | 27.2±5.7 | 27.0±5.4 | 0.35 |
| Obesity | 406 (28.6) | 314 (27.0) | 0.36 |
| Hypertension before pregnancy | 26 (1.8) | 16 (1.4) | 0.43 |
| Family history of diabetes | 489 (33.0) | 400 (34.2) | 0.51 |
| Parity, number of children | 2.3±1.3 | 2.3±1.3 | 0.95 |
| **HIP status** | | | 0.15 |
| eGDM | 20.1 | 31.2 |  |
| GDM | 74.4 | 61.1 |  |
| DIP | 5.5 | 6.5 |  |
| **History of HIP** |  |  | 0.12 |
| 1^st^ child | 477 (32.2) | 368 (31.5) |  |
| no | 784 (52.9) | 601 (51.5) |  |
| yes | 220 (14.9) | 199 (17.0) |  |
| **History of macrosomia** |  |  | 0.67 |
| 1^st^ child | 477 (32.2) | 368 (31.5) |  |
| no | 921 (62.2) | 731 (62.6) |  |
| yes | 83 (5.6) | 69 (5.9) |  |
| **History of fetal death** |  |  | 0.39 |
| 1^st^ pregnancy | 289 (19.5) | 218 (18.7) |  |
| no | 1150 (77.7) | 911 (78.0) |  |
| yes | 42 (2.8) | 39 (3.3) |  |

HIP: hyperglycemia in pregnancy; eGDM: early-diagnosed gestational diabetes mellitus; GDM: gestational diabetes mellitus; DIP: diabetes in pregnancy
