# Supplementary material for: Impact of experiencing multiple vulnerabilities on fetal growth and complications in women with hyperglycemia in pregnancy
Source: BMC Pregnancy Childbirth. 2023 Oct 18;23:740. doi: 10.1186/s12884-023-06048-9 (PMC10585815; doi:10.1186/s12884-023-06048-9)
Supplement: Supplementary file 2 — Additional file 2. [file 12884_2023_6048_MOESM2_ESM.docx]

Additional file 1: EPICES score (**E**valuation de la **P**récarité et des **I**négalités de santé dans les **C**entres d’**E**xamens de **S**anté - Evaluation of Deprivation and Inequalities in Health Examination Centres)

| **Questions** | **Score** |  |
| --- | --- | --- |
|  | **Yes** | **No** |
| 1. Do you sometimes meet with a social worker (welfare worker, educator)? | 10.06 | 0 |
| 2. Do you have complementary health insurance (mutual insurance)? | -11.83 | 0 |
| 3. Do you live as a couple? | -8.28 | 0 |
| 4. Are you a homeowner or will you be one in the near future? | -8.28 | 0 |
| 5. Are there periods in the month when you have real financial difficulties in facing you needs (food, rent, electricity)? | 14.80 | 0 |
| 6. Have you participated in any sports activities in the last 12 months? | -6.51 | 0 |
| 7. Have you gone to any shows (cinema, theatre) in the last 12 months? | -7.10 | 0 |
| 8. Have you gone on holiday during the past 12 months? | -7.10 | 0 |
| 9. Have you seen any family members in the past six months (other than your parents or children)? | -9.47 | 0 |
| 10. Did you have difficulties (financial, family or health), is there anyone around you who could take you in for a few days? | -9.47 | 0 |
| 11. Did you have difficulties (financial, family or health), is there anyone around you who could help you financially (material aid such as lending you money)? | -7.10 | 0 |
| Intercept | 75.14 | 0 |
